# Editorial: Neuronal ceroid lipofuscinosis: A multidisciplinary update

**DOI:** 10.3389/fneur.2022.1083113

**Published:** 2022-11-16

**Authors:** Alessandro Simonati, Ruth Elizabeth Williams, Angela Schulz

**Affiliations:** ^1^Department of Surgery, Dentistry, Paediatrics and Gynaecology, University of Verona School of Medicine, Verona, Italy; ^2^Department of Children's Neuroscience, London, United Kingdom; ^3^Department of Pediatrics, University Medical Center Eppendorf-Hamburg, Hamburg, Germany

**Keywords:** neuronal ceroid lipofuscinosis, genetics, epidemiology, ethics, treatment, experimental models

Eleven papers and fifty-one authors from seven countries have contributed to the Research Topic *Neuronal Ceroid Lipofuscinosis: a Multidisciplinary Update*. Both clinical and research issues have been addressed in this collection of articles. The first paper provides a broad introduction and subsequent articles cover epidemiology and genetics, diagnosis, natural history studies, treatment and ethical implications of novel therapies, cardiac involvement in the later stages of disease and the underlying pathological mechanisms.

The state-of-art in the field of childhood NCLs was described from a number of perspectives in the first review paper of the series (Simonati and Williams). Following a brief historical survey, a clinically-oriented approach was used to describe how the early symptoms and signs represent topographical signatures of the underlying brain dysfunction and may provide clues helping clinicians to reach a conclusive NCL diagnosis rapidly. The paper goes on to document advances in NCL research and the contributions of different experimental models to enhance knowledge of the pathogenic mechanisms underlying cellular pathology in this group of diseases. Lastly, translation of experimental data into novel therapeutic approaches and the importance of symptomatic treatments, which remain the main available therapeutic approaches, were outlined.

The world-wide distribution of NCL was emphasized by the retrospective epidemiological study from South America and the Caribbean region, in which CLN2, CLN6, and CLN3 disorders were identified as the most common NCL types in those regions (Guelbert et al.). The authors have stressed that synergy between health providers, parent support organizations and the pharmaceutical industry have accelerated the use of modern diagnostic procedures.

The significance of the advances in genetic studies in NCL was discussed in the review article by Gardner and Mole which focuses on the genetic basis of phenotypic heterogeneity (Gardner and Mole). Since the discovery of the first causative genes, more than 530 mutations have been identified across 13 NCL genes. Increasing numbers of variant disease phenotypes are being described. Identification of phenotypic heterogeneity combined with the genetic background of each patient is necessary in order to facilitate individually tailored precision medicine in order to modify disease progression in the approaching genomic medicine era.

Based on their own direct experience, which led FDA and EMA Regulatory Agencies to consider natural-history controls valid for the evaluation of efficacy in experimental therapies for CLN2 disease, Nickel and Schulz discuss the importance of collecting natural history data in clinical settings for different purposes, including to advance drug development. The most important requirements of a valid natural history disease registry compliant with data protection and sharing policies, are described. The process of providing high quality quantitative natural history data in a cohort of longitudinally assessed CLN2 disease patients is reported.

The focus of a mini review by Bartsch and Storch is the deterioration and loss of vision caused by progressive retinal degeneration. The therapeutic benefits of treating retinal dystrophies with gene-based approaches (CLN3 and CLN6 mouse models and CLN5 sheep model) and with ocular enzyme replacement therapies (CLN2 and CLN10 mouse models), has led to a clinical trial enrolling CLN2 patients to test the efficacy of intravitreal ERT. The long-term effects of these therapeutic interventions remain to be evaluated.

Ethical issues in care and treatment are the topic of a paper which reflects the long personal clinical experience in the field of this author (Kohlschütter). He identifies two main topics, the first relates to the care of individual patients affected with dementia at a young age, the use of life-prolonging measures and the planning for the end of life, the second refers to new experimental treatments and the awareness that such approaches carry the risk of prolonged survival with poor quality of life. The paper gives examples experienced by the author which offer insights for the “critical thinking” of readers. The issues encountered in caring for patients affected by NCL, but may well be common to other rare neurodegenerative diseases of childhood.

The importance of neurophysiological tools to describe disease evolution and supporting early diagnosis of NCL patients was reviewed through a careful analysis of their characteristics in several NCL types (Trivisano et al.). Authors outline how EEG and (to a less extent) evoked potentials can promptclinicians to obtain a molecular diagnosis in the early phase of any NCL form, which will help to direct patients to appropriate targeted treatments (when available) efficiently.

Reaching an early diagnosis was the aim of a nationwide screening project in Spain amongst children whose early clinical features were consistent with CLN2 (Rodrigues et al.). It used an enzymatic assay of TPP1 activity in dry blood spots, carried out through pediatricians. Authors describe the test as easy to perform, inexpensive and reliable and conclude that such a test may contribute to early delivery of ERT in this condition.

The next two articles concern cardiac involvement in CLN3 disease. In their report describing a case series of six, Handrup et al. state that pacemaker implantation is safe and positively impacts on quality of life of patients because of the presence of early cardiac conduction disorders and later left ventricular hypertrophy. The ethical implications of such therapeutic option are also commented upon. In a single case report the accuracy of comprehensive cardiac MRI findings are reported in a CLN3 disease patient. Authors recommend the advantages of cardiac MRI both for early diagnosis of cardiac complications of NCL and its value in monitoring the effects of emerging CLN3 therapies on the myocardium (Todiere et al.).

In the only review article related to experimental models of NCLs, Takahashi et al., describe the current knowledge on the role of the different glia components (astrocytes, microglia, oligodendrocytes) in brain homeostasis. They go on to focus on the most up-to-date understanding of glial pathologies and their contribution to the pathogenesis of NCLs: they highlight some of the associated challenges that require further research as obtained by their studies using genetically modified mouse models. The emerging evidence of glial dysfunction questions the traditional “neuron-centric” view of NCLs, and would suggest that directly targeting glia in addition to neurons could lead to better therapeutic outcomes.

In summary, this series of articles is drawn from world experts in NCL. It brings together basic science and new clinical knowledge, while considering the ethical implications of recent progress on individual patients, families and their physicians and clinicians.

## Author contributions

All authors listed have made a substantial, direct, and intellectual contribution to the work and approved it for publication.

## Funding

This work was funded by the Italian Ministry for Universities and Research (MUR) and Fondazione Mariani (grant National Network on Rare Neuropediatric Diseases) to ASi is acknowledged.

## Conflict of interest

The authors declare that the research was conducted in the absence of any commercial or financial relationships that could be construed as a potential conflict of interest.

## Publisher's note

All claims expressed in this article are solely those of the authors and do not necessarily represent those of their affiliated organizations, or those of the publisher, the editors and the reviewers. Any product that may be evaluated in this article, or claim that may be made by its manufacturer, is not guaranteed or endorsed by the publisher.

